# Chitinase mRNA Levels by Quantitative PCR Using the Single Standard DNA: Acidic Mammalian Chitinase Is a Major Transcript in the Mouse Stomach

**DOI:** 10.1371/journal.pone.0050381

**Published:** 2012-11-21

**Authors:** Misa Ohno, Kyoko Tsuda, Masayoshi Sakaguchi, Yasusato Sugahara, Fumitaka Oyama

**Affiliations:** Department of Applied Chemistry, Kogakuin University, Hachioji, Tokyo, Japan; University of Tübingen, Germany

## Abstract

Chitinases hydrolyze the β-1-4 glycosidic bonds of chitin, a major structural component of fungi, crustaceans and insects. Although mammals do not produce chitin or its synthase, they express two active chitinases, chitotriosidase (Chit1) and acidic mammalian chitinase (AMCase). These mammalian chitinases have attracted considerable attention due to their increased expression in individuals with a number of pathological conditions, including Gaucher disease, Alzheimer’s disease and asthma. However, the contribution of these enzymes to the pathophysiology of these diseases remains to be determined. The quantification of the Chit1 and AMCase mRNA levels and the comparison of those levels with the levels of well-known reference genes can generate useful and biomedically relevant information. In the beginning, we established a quantitative real-time PCR system that uses standard DNA produced by ligating the cDNA fragments of the target genes. This system enabled us to quantify and compare the expression levels of the chitinases and the reference genes on the same scale. We found that AMCase mRNA is synthesized at extraordinarily high levels in the mouse stomach. The level of this mRNA in the mouse stomach was 7- to 10-fold higher than the levels of the housekeeping genes and was comparable to that the level of the mRNA for pepsinogen C (progastricsin), a major component of the gastric mucosa. Thus, AMCase mRNA is a major transcript in mouse stomach, suggesting that AMCase functions as a digestive enzyme that breaks down polymeric chitin and as part of the host defense against chitin-containing pathogens in the gastric contents. Our methodology is applicable to the quantification of mRNAs for multiple genes across multiple specimens using the same scale.

## Introduction

Chitin, a linear polymer of β-1-4-linked *N*-acetyl-D-glucosamine, is the second most abundant polysaccharide found in nature. It functions as a major structural component of fungi, crustaceans, and insects but is not found in mammals [1]. Chitinases hydrolyze the β-1-4 glycosidic bonds of the chitin polymer. Although mammals do not produce chitin or its synthase, they express two active chitinases, chitotriosidase (Chit1) and acidic mammalian chitinase (AMCase) [2], [3].

The Chit1 level is markedly elevated in the plasma of patients with Gaucher disease, an autosomal recessive lysosomal storage disorder [4]. Chit1 was the first mammalian chitinase to be purified and cloned [5], [6]. A recessively inherited deficiency in Chit1 activity is commonly observed in Caucasians [7]. AMCase was discovered because of its compensatory role and was named for its acidic pH optimum [8]. These mammalian chitinases are regarded as part of the host defense mechanism against chitin-containing pathogens and parasites [3], [9].

Both Chit1 and AMCase are secreted proteins with molecular weights of approximately 50 kDa. Both proteins contain an N-terminal catalytic domain, a hinge region, and a C-terminal chitin-binding domain [6], [8]. Mouse AMCase shows sequence homology to Chit1, with an identity of 52% and a similarity of 60% [10]. Despite these structural similarities, these enzymes significantly differ with respect to their enzymatic behavior at acidic pH. AMCase shows a marked pH optimum at pH 2 and a less evident optimum at pH 4–7 [8], whereas Chit1 shows only a broad pH optimum at around pH 5 [5], [11].

Mammalian chitinases have attracted considerable attention due to their increased expression in individuals with different pathological conditions. Chit1 is increased in individuals with Gaucher disease [4], chronic obstructive pulmonary disease (COPD) [12], and Alzheimer’s disease [13] and in smokers [14]. AMCase expression and activity are also upregulated during allergic airway responses in mouse models of asthma [15]. In addition, polymeric chitin induces AMCase expression and the recruitment of immune cells associated with allergy and asthma [16]. These results strongly suggest that chitinolytic enzymes play important roles in many pathophysiological conditions. However, the contribution of these enzymes to the pathophysiology of these diseases remains to be determined.

The quantification of the Chit1 and AMCase mRNA levels and the comparison of those levels with the levels of well-known reference genes are important steps in gaining insight into the *in vivo* regulation of mammalian chitinases. Recently, real-time RT-PCR has been used to quantify mRNA levels in many gene expression studies [17]–[20] because this method is sufficiently sensitive to detect mRNA from even a single cell. Real-time PCR commonly involves the normalization of the expression levels of the gene of interest with those of the housekeeping genes that are thought to be consistently expressed in all of the samples. However, this quantification method fails to compare the levels of the different gene transcripts on the same scale.

In the present investigation, we developed a quantitative real-time RT-PCR system that uses standard DNA produced by ligating the cDNA fragments of the target genes. This system enabled us to quantify and compare the expression levels of the chitinases and the reference genes on the same scale. Our results indicate that AMCase is a major transcript in the mouse stomach, suggesting that the corresponding protein functions as a digestive enzyme that breaks down chitin-containing foods and as part of the host defense against chitin-containing pathogens in the gastric contents.

## Materials and Methods

### RNA, RNA Isolation and cDNA Preparation

The Mouse Total RNA Master Panel (Clontech Laboratories) was used to examine the tissue distribution of transcripts. We analyzed four different embryonic stages and eight adult tissues. In addition, RNA was isolated from the lungs and stomachs of 3-month-old male mice. C57BL/6J mice (CLEAR Japan) were bred at the RIKEN Brain Science Institute Animal Facility. All animal experiments were performed in compliance with the institutional guidelines. The protocol was approved by the Committee on the Ethics of Animal Experiments of the RIKEN Brain Science Institute (Approval No. H19-2B013). All surgery was performed by using diethyl ether as an anesthetic, and all efforts were made to minimize suffering. Those tissues for mRNA preparation were provided by Drs. Miyazaki and Nukina at RIKEN Brain Science Institute. Total RNA was prepared from lungs and stomachs using TRIzol Reagent (Invitrogen) according to the manufacturer’s instructions. To remove the trace amounts of contaminating genomic DNA, the total RNA samples were treated with RQ1 RNase-Free DNase (Promega) according to the manufacturer’s recommended protocol. The concentrations of the nucleic acids were determined by measuring the absorbance at 260 nm using a BioPhotometer Plus (Eppendorf). Each of the total RNA samples (3 µg) was subjected to reverse transcription with random hexamers as primers. The reaction mixture (15 µl) contained the enzyme buffer [50 mM Tris-HCl (pH 8.3), 75 mM KCl, and 3 mM MgCl_2_], 100 ng of random hexamers, 10 mM dithiothreitol, and 0.5 mM deoxynucleotide triphosphates (dNTPs). After heating the solution to 60°C for 5 min and incubating the mixture at 37°C for 5 min, 200 U of recombinant murine leukemia virus reverse transcriptase (Invitrogen) was added, and the mixture was incubated at 37°C for 45 min. The reverse transcription was terminated by heating to 95°C for 5 min.

### Real-time PCR

Primers for real-time RT-PCR were designed based on Primer Express Software (Applied Biosystems) and were synthesized commercially (Sigma-Genosys, Sigma-Aldrich). The PCR reactions were performed in a final volume of 13 µl containing 2 × SYBR Green Master Mix (Brilliant II SYBR Green QPCR Master Mix, Agilent), 2.7 ng of mouse cDNA or appropriate dilutions of the external standards (see below), and the appropriate concentrations of the primers for the chitinases, pepsinogen C, glyceraldehyde-3-phosphate dehydrogenase (GAPDH) or β-actin. Standard real-time PCR conditions for the system (Mx3005P, Agilent) were used: an initial denaturation and polymerase activation step for 10 min at 95°C, followed by 40 cycles of denaturation at 95°C for 1 min, 55°C for 30 sec, and 72°C for 1 min. Melting curves were generated after amplification. The PCR products were electrophoresed on a 10% polyacrylamide gel and analyzed using the Luminescent Image Analyzer (ImageQuant LAS 4000, GE Healthcare). The PCR solution was treated with ExoSAP-IT (USB Products) according to the manufacturer’s instructions to eliminate unincorporated primers and dNTPs, and the products were sequenced using the ABI PRISM Big-Dye Terminator v3.1 Cycle Sequencing Kit and a 3130 Genetic Analyzer (Applied Biosystems). The nucleotide sequences of the primers selected for the real-time PCR are shown in **Table S1**.

### Construction of the Standard DNA

The standard template cDNA (913 bases) for the quantification of transcript levels by real-time PCR was constructed as follows. The cDNA fragments covering the PCR-target region plus 9–120 bases of the flanking regions of AMCase, Chit1, pepsinogen C, GAPDH, and β-actin were amplified from mouse stomach cDNA by PCR using KOD Plus DNA polymerase (Toyobo) and oligonucleotides primer containing the restriction sites of *Bgl*II, *Xho*I, *Pst*I, or *Not*I (at 5′- and 3′- ends) according to the manufacturer’s protocol. The forward and reverse primers are listed in **Table S2**. The PCR products were purified using the Wizard SV Gel and PCR Clean-Up System (Promega) and then digested with the corresponding restriction enzymes. The DNA fragments were purified using agarose gel electrophoresis and the Clean-Up System and then ligated together with T4 DNA ligase (Toyobo). The ligated fragments were amplified using the forward primer 5′-GTGGATTCTGTGCCGACAAAGCAGATGGCC-3′ and the reverse primer 5′-TGGGTACATGGTGGTACCACCAGACAGCAC-3′ with KOD Plus DNA polymerase. 3′-dA was added to the amplified DNA using Takara Taq HS (Takara Bio), and the product was purified by gel electrophoresis as described above. The resulting DNA was cloned into the pGEM-T Easy vector (Promega) through TA cloning, according to the manufacturer’s instructions. The nucleotide sequence of the resulting plasmid was confirmed by sequencing. The linearized multigene-containing DNA fragment was prepared by reamplification from the plasmid DNA with the same primers by PCR using KOD Plus DNA polymerase. The fragments were purified and quantified as described above and used as the standard DNA.

### Preparation of Five cDNAs Covering the Entire Coding Region

To validate our absolute real-time PCR method, we prepared full coding cDNAs by PCR using the primer sets listed in **Table S3**. Five cDNAs covering the entire coding regions of two chitinases (Chit1 and AMCase) and the reference genes (GAPDH, β-actin and pepsinogen C) were amplified by PCR from mouse stomach cDNA using KOD Plus DNA polymerase and were subcloned into the pGEM-T Easy vector through TA cloning, as described above. The sequences of the cDNAs were verified by sequencing, as described in **Figure S1**. The subcloned fragments were reamplified from the plasmid DNAs with the same primers and were then used as the entire coding region cDNAs.

### Standard Curves

The molar concentration of the multigene-containing DNA standard was calculated based on the concentration and the molecular weight. Serial dilutions were prepared starting with the standard template concentration, which yielded a Ct of approximately 13 (Ct = fractional threshold cycle value). The standard DNA was subjected to 10-fold serial dilutions, ranging from 10^0^ to 10^7^ molecules, and the aliquots were kept frozen at −20°C until use.

### Quantification of mRNA by Real-time PCR using the Standard Curves or the ΔΔ Ct Method

Each sample was amplified in triplicate, and each experiment was repeated at least two times. Using the standard curve, the numbers of Chit1, AMCase, GAPDH, β-actin, and pepsinogen C mRNA molecules were extrapolated automatically by using the MxPro QPCR Software version 4.10 (Agilent). All values were expressed as molecules per 10 ng of total RNA. In some instances, the values were normalized against the levels of GAPDH or β-actin mRNA.

For evaluating our present methodology, we also employed the ΔΔ Ct method [21], which requires the measurement of the cycle threshold (Ct) of target genes. Ct values were calculated by the MxPro QPCR Software using GAPDH or β-actin as a normalizer.

## Results

### Establishment of the Real-time PCR Assay

**Figure 1:**
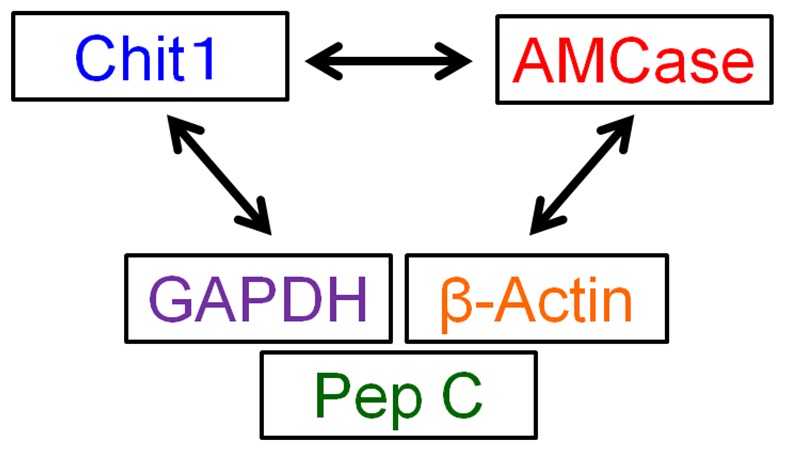
Strategy for comparing the gene expression levels of five genes. The expression levels of the Chit1 and AMCase genes were compared. To evaluate the chitinase levels, we used two housekeeping genes, GAPDH and β-actin, which are constitutively expressed at high levels in most tissues and cells. In addition, we chose pepsinogen C, which is a major component of the gastric mucosa, as a reference gene for the stomach tissues. Using these three reference genes, we evaluated the gene expression levels of Chit1 and AMCase in mouse tissues.

The establishment of a reliable method for the measurement of the expression of chitinase transcripts is an important step in investigating the significance of mouse chitinases. Real-time RT-PCR is, at present, the most sensitive quantitative method for the detection of both low-abundance mRNAs and abundant transcripts. We first compared the gene expression levels of the Chit1 and AMCase genes (see Figure 1). To evaluate the chitinase levels, we used two housekeeping genes, GAPDH and β-actin, as reference genes because they are constitutively expressed at high levels in most tissues and cells [22]. In addition, we chose pepsinogen C (also known as progastricsin) as a reference gene in the stomach. Pepsinogen C is an aspartic protease that functions as a digestive enzyme and is produced in the stomach. This enzyme constitutes a major component of the gastric mucosa [23]. Using these three reference genes, we evaluated the gene expression of Chit1 and AMCase in mouse tissues (Figure 1).

**Figure 2:**
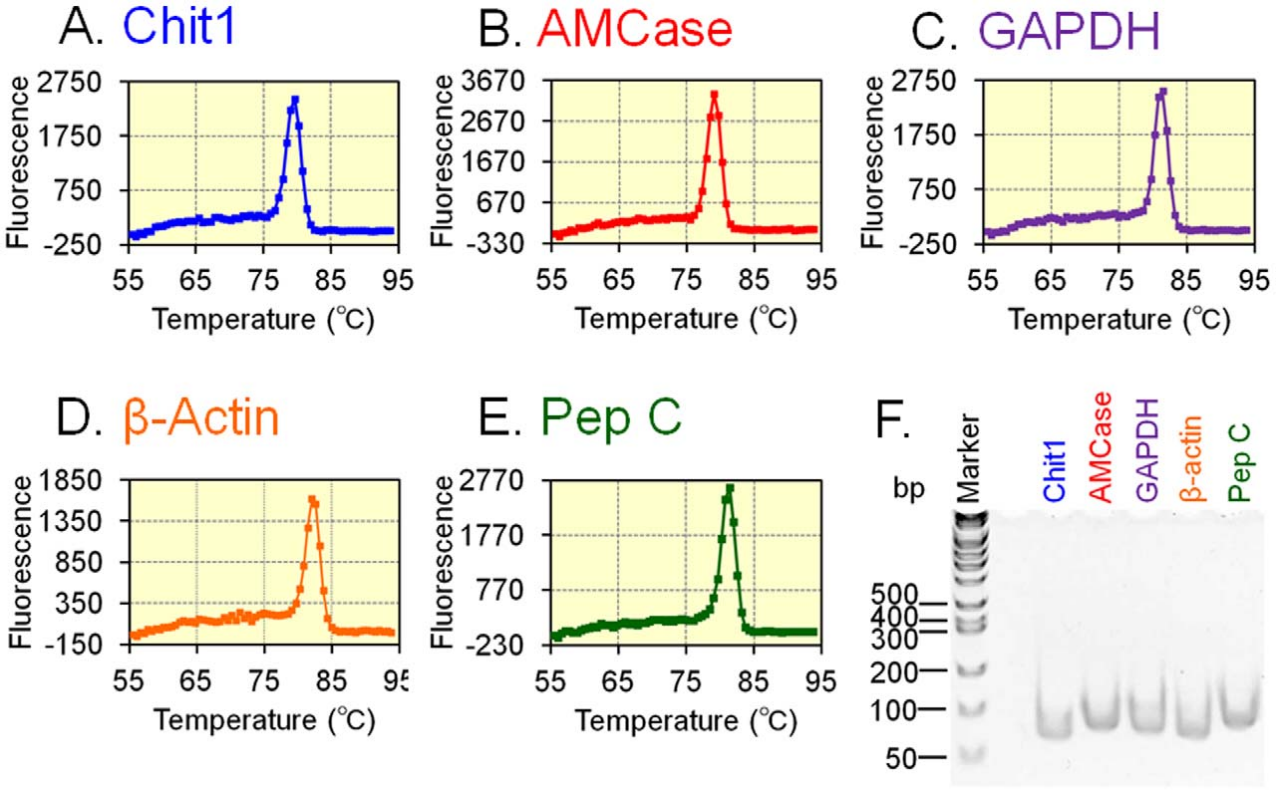
Evaluation of primer suitability for real-time PCR. PCR primers were selected based on whether they gave one melting temperature (A–E) and a single PCR product on a 10% polyacrylamide gel (F). To verify the specificity of the primers, the dissociation curves of the PCR products for five genes generated using mouse tissue cDNA mixture were examined. The PCR products were analyzed on a 10% polyacrylamide gel, followed by ethidium bromide staining. The nucleotide sequences of the PCR products were confirmed.

**Figure 3:**
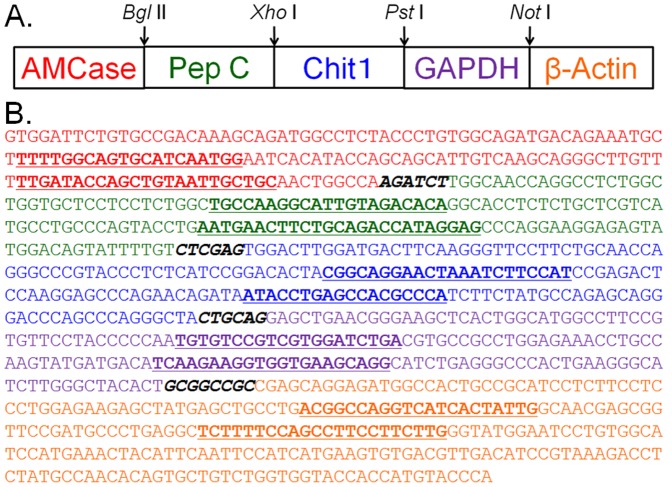
Construction of standard template DNA. A. Schematic representation of the standard template DNA used for real-time PCR. The target fragments of the AMCase, pepsinogen C, Chit1, GAPDH and β-actin cDNAs with flanking sequences and restriction sites were amplified and ligated in a one-to-one ratio into a DNA fragment, which was then cloned into a cloning vector. The linearized standard template DNA was amplified from the plasmid DNA and used as the standard DNA. B. Nucleotide sequence of the standard template DNA. The template DNA, 913 nucleotides long, contained five cDNA fragments (shown in different colors) covering the PCR-target region (shown in bold underline) plus 9–120 bases of the flanking regions and containing restriction sites for *Bgl*II, *Xho*I, *Pst*I, or *Not*I (bold italic).

We first designed several sets of primers for quantitative PCR and evaluated their suitability based on whether they gave single products, as reflected by a single melting temperature (Tm) and a single band on a 10% polyacrylamide gel. The nucleotide sequences of the products were also verified. To examine the specificity of the primers, each of the PCR products was amplified under real-time PCR conditions by using a mouse tissue cDNA mixture consisting of tissues from four embryonic stages and eight adult tissues and was assayed in combination with different methods. As shown in Figure 2A–E, only one peak appeared in the dissociation curves for Chit1 (Tm = 79.7°C), AMCase (Tm = 79.1°C), GAPDH (Tm = 81.4°C), β-actin (Tm = 82.0°C) and pepsinogen C (Tm = 81.4°C). At the end of the PCR, we analyzed the products on a 10% polyacrylamide gel. Gel electrophoresis showed clear single bands at the expected sizes of Chit1 (69 bp), AMCase (81 bp), GAPDH (77 bp), β-actin (71 bp) and pepsinogen C (82 bp) (Figure 2F). The nucleotide sequences of the PCR products were determined directly as described in the Materials and Methods section. We confirmed that the PCR products were amplified from the target cDNAs (see Figure 3B). These results indicate that the PCR products are specific amplicons from the target cDNAs and that mispriming is negligible under our experimental conditions.

### Construction of the Standard Template DNA for the Quantification and Comparison of Gene Expression Among Five Genes

We sought to compare the expression levels of two chitinases and three reference genes on the same scale (Figure 1). For this purpose, we set up a quantitative real-time PCR system for which a standard template was necessary for accurate quantification (Figure 3A). We constructed a standard template DNA for real-time PCR by ligating the five target fragments in a one-to-one ratio, and then this ligated DNA fragment was cloned into the plasmid vector as described in the Materials and Methods section (Figure 3A). The 913-nucleotide-long template DNA contained five cDNA fragments covering the PCR-target region plus 9–120 bases of the flanking regions and contained the restriction sites of *Bgl*II, *Xho*I, *Pst*I and *Not*I (see details in Figure 3B).

### Validation of the Standard Curve and the Quantitative Real-time PCR System

**Figure 4:**
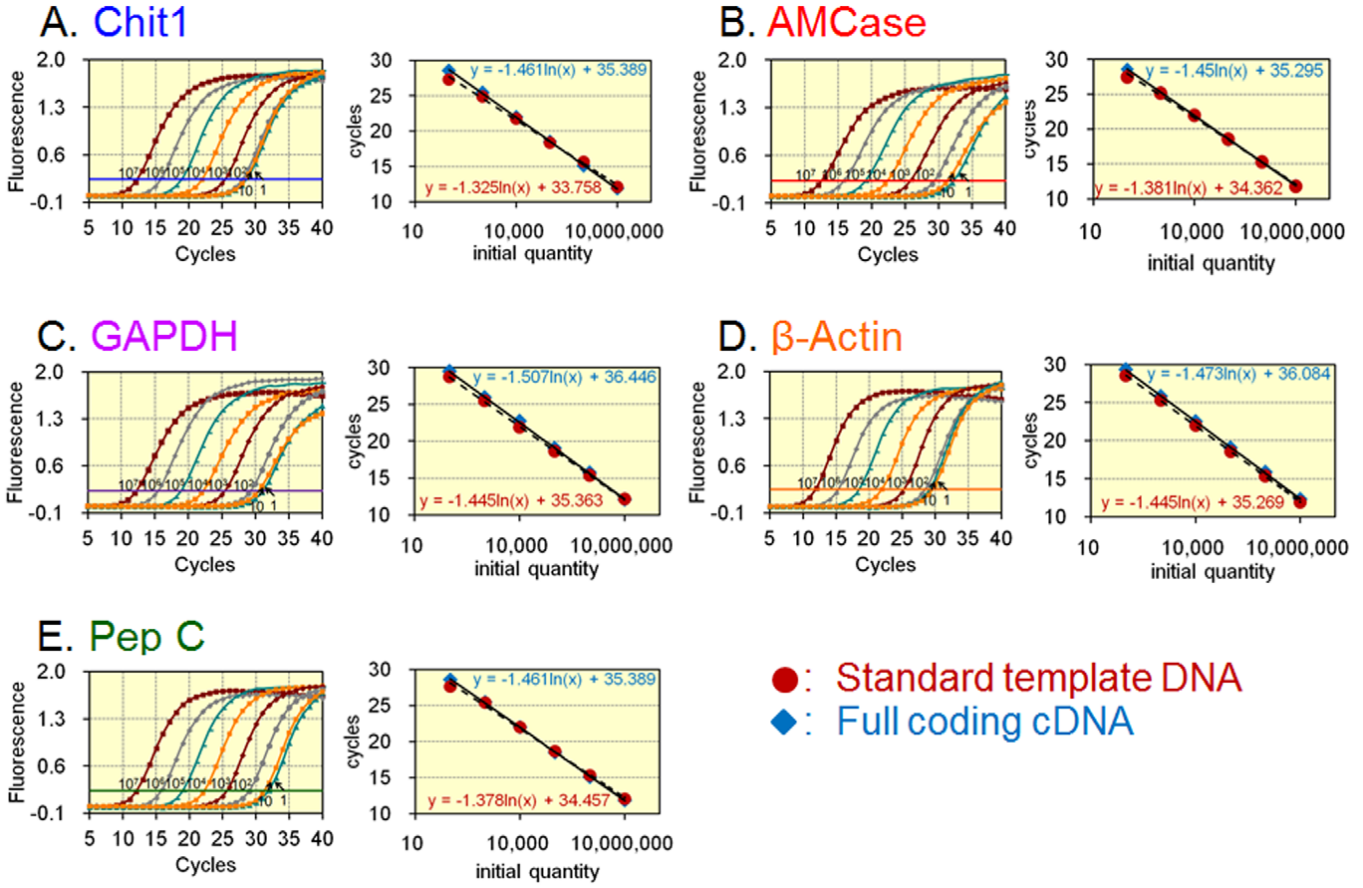
Establishment and validation of a quantitative real-time PCR system using the standard template DNA. The checked DNAs are as follows: A, Chit1; B, AMCase; C, GAPDH; D, β-actin; E, pepsinogen C. (Left) Real-time PCR quantification of each 10-fold serial dilution of the standard template DNA using primer pairs targeting each gene. (Right) The standard curves by using the standard template DNA containing the five cDNA fragments (brown closed circles). In addition, the quantification of full coding cDNAs was carried out using primer pairs for each gene. The target cDNA was amplified from a dilution of the full coding cDNA with a known concentration (see **Figure S2**) and subsequently analyzed as an unknown sample (blue closed rhombuses). Equal quantities were observed for each tested dilution of the standard curve and full coding cDNA.

The quantification of both the chitinases and the reference mRNAs relies on standard curves. Serial dilutions of the standard template DNA were used to construct a standard curve to compare and evaluate the real-time RT-PCR quantification strategies that were used to analyze the five mRNAs. Each standard curve was generated using 10-fold serial dilutions of the standard DNA and the five different primer pairs (Figure 4A–E, left). Exponential amplification was maintained over a wide range of cycles, yielding a dynamic range of seven orders of magnitude (Figure 4A–E, left). By using the standard template DNA containing the five cDNA fragments, equal quantities can be assigned to all five genes in each dilution use to construct the standard curves (Figure 4A–E, right).

To test the absolute equality of the curves, a known concentration of the full coding cDNA was amplified and subsequently analyzed as an unknown sample. This assay was performed to verify that each tested dilution resulted in the expected quantity. As shown in Figure 4A–E, right, equal quantities were observed for each tested dilution used to construct the standard curve (see **Figure S2**). The quantification of low-abundance transcripts and abundant transcripts allows us to validate the sensitivity and reliability of real-time PCR, indicating that our real-time PCR method offers a large dynamic range of quantification, high accuracy, and high sensitivity (Figure 4A–E, right). Thus, our real-time PCR method provides reliable values for two chitinase genes and for reference genes on the same scale.

### Expression of Chit1 and AMCase in Mouse Tissues

**Figure 5:**
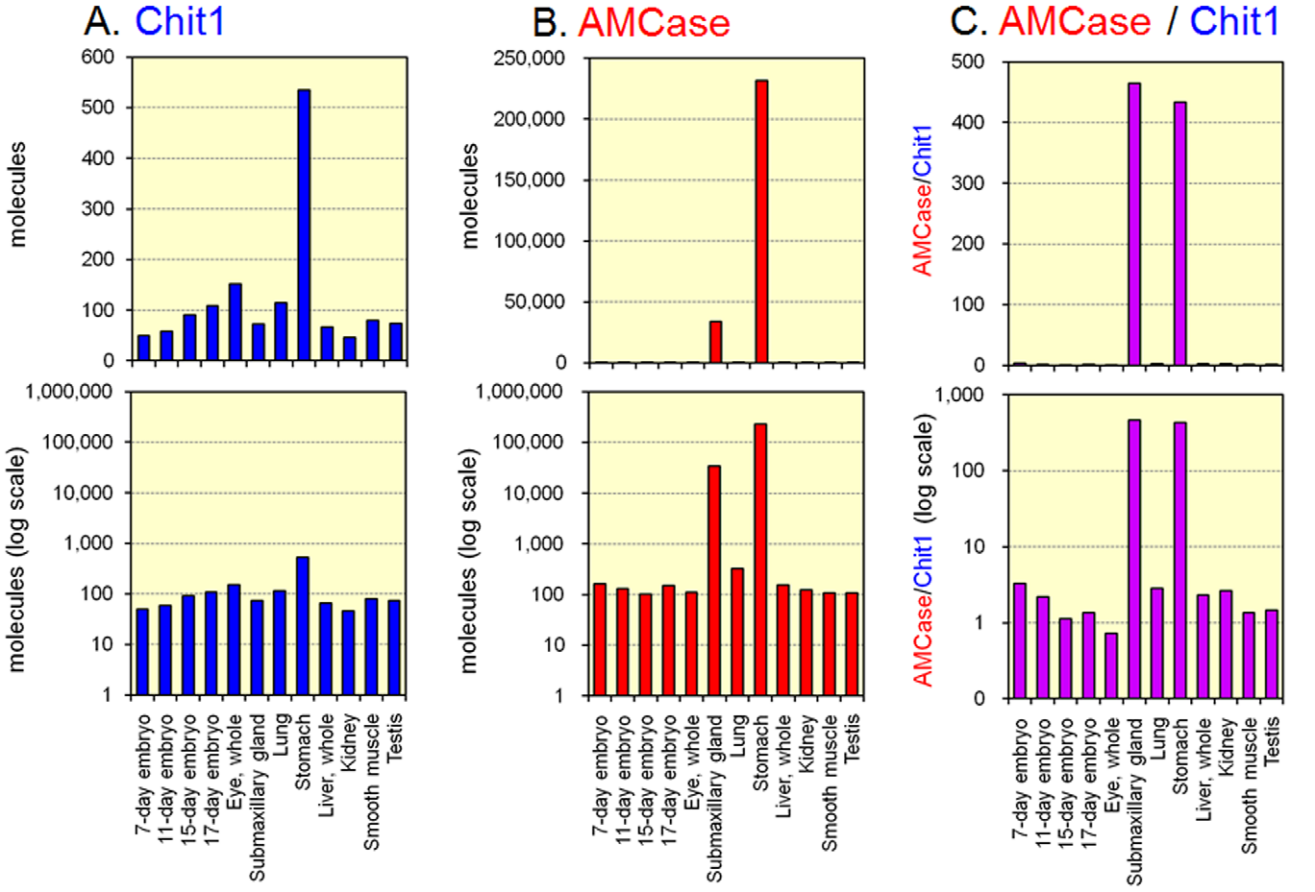
Expression of Chit1 and AMCase mRNAs in mouse tissues. Quantification of Chit1 (A) and AMCase (B) mRNAs in mouse tissues. Both chitinases were quantified by real-time PCR using the standard template DNA. All values obtained were expressed as molecules per 10 ng of total RNA. C. Ratios of AMCase to Chit1. All mRNA copy numbers were derived based on the same standard dilutions. The upper panel indicates the actual number, whereas the lower panel shows the logarithm of the values.

**Figure 6:**
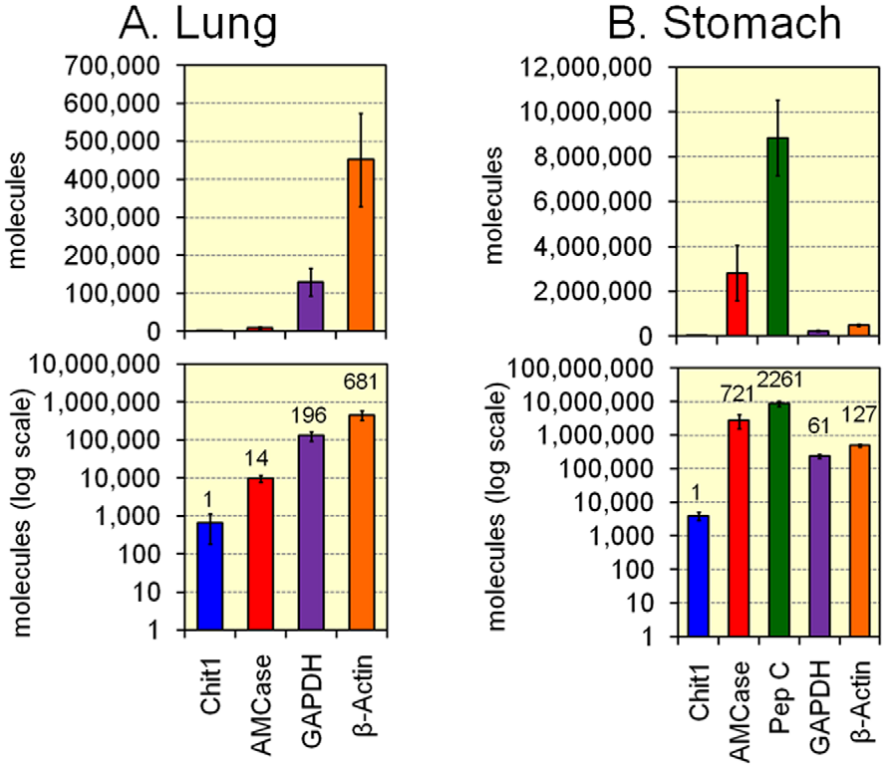
Analysis of Chit1 and AMCase mRNAs and reference gene mRNAs in lung and stomach tissues. The expression levels of the five genes determined using the cDNAs prepared from lung (A) or stomach (B) tissues from 3-month-old mice (n = 5) were quantified by real-time PCR. The upper panel indicates the actual value, whereas the lower panel shows the logarithm of each value.

To study the *in vivo* regulation of Chit1 and AMCase gene expression, total RNA samples extracted from four embryonic stages and from various adult tissues, were analyzed with a quantitative real-time PCR assay using the single standard DNA (Figure 3). The results were expressed as molecules per 10 ng of total RNA (Figure 5 and Figure 6). Both the Chit1 and AMCase mRNAs were widely expressed in mouse tissues (Figure 5A and 5B). Clear tissue specificities were observed in the expression patterns of both chitinase mRNAs. High levels of Chit1 mRNA were detected in the mouse stomach (Figure 5A, upper panel), followed by the eyes and the lung. Chit1 mRNA was detected at low, but readily detectable, levels in other tissues (Figure 5A, lower panel). AMCase mRNA was predominantly detected in the stomach, followed by the submaxillary gland (Figure 5B, upper panel), but was also present in other tissues (Figure 5B, lower panel).

We then compared the ratios of AMCase to Chit1. The copy number of each mRNA was determined using the same standard dilutions. We found that the stomach and the submaxillary gland predominantly expressed AMCase mRNA (Figure 5C, upper panel). The other tissues examined in this study produced more AMCase than Chit1, and Chit1 was prevalent only in the eyes (Figure 5C, lower panel).

We next examined the relative quantification in the original data presented in Figure 5, which was expressed as molecules per 10 ng of total RNA, using housekeeping genes as described in Materials and methods. The results were shown as Supporting Information in **Figure S3** (data was normalized by GAPDH) and **Figure S4** (normalized by β-actin). Then, we analyzed data shown in Figure 5 by the ΔΔ Ct method [21] and shown as Supplementary Information in **Figure S5** (normalized by GAPDH) and **Figure S6** (normalized by β-actin). When comparing the relative expression detected by the present absolute and relative quantification methods, there was no significant difference among the data except for Chit1 mRNA expression, which was most abundantly expressed in the eyes when normalized by β-actin (see Figure 5, **Figure S3** and **Figure S4**). Similar results were also obtained by relative quantification using the ΔΔ Ct method [21] (see Figure 5, **Figure S5** and **Figure S6**). However, we could not directly evaluate mutual expression levels of Chit1 and AMCase when analyzed by the ΔΔ Ct method.

### Analysis of Chit1, AMCase, Pepsinogen C, GAPDH, and β-actin in Lung and Stomach Tissues

Many studies have been carried out on the pathophysiology of mammalian chitinases in lung tissues. In this study, we showed that AMCase mRNA is predominantly expressed in mouse stomach tissues (Figure 5). We also compared the expression levels of the chitinases and reference genes using the cDNAs prepared from the lung and stomach tissues of 3-month-old mice (n = 5). The quantitative data are shown in Figure 6. When the Chit1 levels were set at 1.0, the relative expression levels of cDNAs were AMCase, 14; GAPDH, 196; and β-actin, 681 in the mouse lung tissue (Figure 6A). This result indicates that lung tissues express more AMCase than Chit1, although the AMCase expression level was lower than that of the two housekeeping genes.

In stomach tissues, when the Chit1 level was set at 1.0, the relative expression levels were AMCase, 721; pepsinogen C, 2261; GAPDH, 61; and β-actin, 127 (Figure 6B). Both GAPDH and β-actin genes are well-known housekeeping genes and are constitutively expressed at high levels in most tissues and cells. Pepsinogen C (progastricsin) is an aspartic protease that functions as a digestive enzyme and is produced in the stomach. This enzyme is a major component of the gastric mucosa [23]. The expression level of AMCase was much higher than those of GAPDH and β-actin and was comparable to the level of pepsinogen C. These results indicate that stomach AMCase is a major transcript in the gastric mucosa and suggest that this enzyme is likely to play important physiological roles in the stomach.

We also re-examined relative expression of Chit1, AMCase and pepsinogen C shown in Figure 6 using housekeeping genes by present method as described above. The results were shown as Supporting Information in **Figure S7** (normalized by GAPDH) and **Figure S8** (normalized by β-actin). There was no significant difference among Figure 6, **Figure S7** and **Figure S8**.

## Discussion

Both Chit1 and AMCase are thought to aid in the host defense against chitin-containing pathogens [3], [9]. In addition, AMCase is an effector molecule in allergic inflammation. These chitinolytic enzymes play important roles in the pathophysiology of allergic airway responses in mouse models of asthma. Relatively little is known, however, about the *in vivo* mutual expression levels of Chit1 and AMCase. The quantification of mRNAs can generate useful and biomedically relevant information. In this study, we established a quantitative real-time PCR system capable of determining the mRNA levels of two mammalian chitinases and of comparing these levels with those of reference genes using the same scale. Our results indicate that AMCase is predominantly expressed in the mouse stomach.

Quantitative real-time RT-PCR represents an important advance in quantifying mRNA and allows the detection of the expression of a particular gene of interest at the molecular level. There are many reports on the detection of extremely low levels of mRNA using this technology. However, real-time RT-PCR is not a widely used because it still has limitations in quantifying multiple mRNAs using the same scale. We constructed a standard template DNA comprising five cDNA fragments (each ∼200 bases long). These fragments included fragments for two chitinases, one marker and two housekeeping genes and were combined in one-to-one ratios (Figure 3A). GAPDH and β-actin, the most commonly used housekeeping genes, were included as internal controls and references for PCR amplification. In addition, we used pepsinogen C as a marker gene because it is produced at a high level in the stomach [23]. This method allowed the absolute quantification of the number of Chit1 and AMCase mRNA molecules, as well as the number of reference mRNA molecules, per 10 ng of total RNA (mole/10 ng) (Figure 5 and Figure 6). Furthermore we could examine relative quantification of the Chit1 and AMCase mRNA levels using two housekeeping genes, GAPDH and β-actin (Supporting Information **Figure S3** and **Figure S4**).

Relative quantification is easier to perform than absolute quantification because a calibration curve is not necessary. In relative quantitation, mRNA levels of the gene of interest are compared from those of the housekeeping genes [21]. However, this quantification method fails to compare the levels of the different gene transcripts on the same scale. Although our method requires multiple steps associated with the construction of the standard DNA and with the validation processes, this method can provide gene expression data that are directly comparable between genes (Figure 5 and Figure 6). Our real-time PCR provided a large dynamic range of quantification and high sensitivity, and the quantification of low-abundance transcripts allowed us to validate the sensitivity and reliability of our method (Figure 4). This technique is very well suited to the quantification and comparison of mRNA levels across multiple genes using the same scale. Our present method is applicable to biomedical engineering as well as to clinical and practical uses.

In the present investigation, we found that AMCase mRNA is synthesized at extraordinarily high levels in the mouse stomach relative to the levels of housekeeping genes (Figure 5 and Figure 6). The level of AMCase mRNA is comparable to that of pepsinogen C (progastricsin) mRNA, a major component of the gastric mucosa in the stomach (Figure 6B). In addition to the stomach, the submaxillary glands were also found to express large amounts of AMCase (Figure 5). As for synthesizing mammalian chitinase mRNAs, both stomach and submaxillary glands are regarded as notable tissues for producing enormous amount of AMCase in mouse.

Hydrochloric acid is secreted in the stomach, creating acidic conditions for the digestion of proteins by pepsin at approximately pH 2 [23], [24]. Mouse AMCase shows profound acid stability and is most active at pH 2.0, which distinguishes it from other mouse enzymes [8]. The unusual pH stability and dependence of the mouse AMCase on acidic conditions allow the efficient digestion of chitinous materials under acidic conditions. The observation that AMCase is predominantly expressed in the stomach points to its possible function in food processing. Wild mice eat chitin-containing foods such as insects, whereas mice kept in the laboratory eat artificial diets containing dried brewers’ yeast, which also contains chitin in the cell wall. Thus, AMCase may function as a digestive enzyme that breaks down polymeric chitin in the gastric contents.

The highest level of Chit1 was also expressed in the mouse stomach. However, the Chit1 expression level in the stomach was 1/721 that of the AMCase expression level and was lower than the expression levels of two housekeeping genes, GAPDH and β-actin (Figure 6A). In addition, Chit1 does not possess any chitinolytic activity at the pH of gastric juices, which is around pH 2 [5], [11]. It has been shown that Chit1 is produced at sites of near-neutral pH, such as the non-glandular portion of the stomach and the small intestine [25]. Thus, Chit1 does not contribute to chitinase activity in the mouse stomach.

Although AMCase is expressed predominantly in the stomach and submaxillary glands, all other tissues examined in this study expressed low, but detectable, levels of Chit1 and AMCase. The expression levels of Chit1 and AMCase were lower than those of the housekeeping genes in these tissues. Chit1 shows a broad pH optimum at around pH 5 [5], whereas the primary optimal pH of AMCase is pH 2 and the secondary pH optimum is at pH 4–7, with AMCase retaining less than 30% of its activity at pH 2 in this pH range [8]. Both Chit1 and AMCase digest natural chitin and chitosan via endo-chitinase activity and produce chitobiose [8], [26]. Although AMCase is dominant over Chit1 in many mouse tissues, there may not be any significant differences in their chitinolytic activity in mouse tissues.

More AMCase mRNA than Chit1 mRNA was observed in most mouse tissues, except for the eyes. Our results also suggest that gene expression of Chit1 may be developmentally regulated, suggesting that it plays a role in ontogeny. Studying the regulation of the expression of the mammalian chitinases could give insights into the physiological roles of these enzymes. A detailed characterization of the promoter regions of Chit1 and AMCase genes as well as the identification of *cis*- and *trans*-acting factors will be required to understand the selective expression of these enzymes.

Although mammals do not produce chitin or chitin synthase, they are continually exposed to this polymer through exposure to chitin-containing parasites and pathogens. The substrate for mammalian chitinases is presumably environmental chitin, such as that found in fungi or parasitic nematodes. In the lungs, both enzymes can act as part of the host defense mechanism against chitin-containing pathogens, such as environmental mold and mites, that induce airway allergy. In addition, AMCase in the mouse stomach also plays a specific role in food processing.

Data on the pathophysiological function of AMCase in the human system are contradictory [12]–[15], [27]. Using the PCR technology described herein, we have started measuring the gene expression of the two mammalian chitinases, Chit1 and AMCase, in human tissues. Our preliminary analysis indicates that the AMCase is not overexpressed in human stomach (results will be published elsewhere). We will carry out careful comparison the human chitinase mRNA expression levels across different human tissues, which is the prerequisite for better understanding the pathophysiological roles of mammalian chitinases in human tissues.

### Conclusion

We established a quantitative real-time PCR system to evaluate the expression levels of two mammalian chitinases, two housekeeping genes and one stomach marker gene using a laboratory-constructed standard DNA. AMCase is predominantly expressed in mouse stomach, where the AMCase mRNA level is comparable to that of pepsinogen C, a major digestive enzyme in the gastric juice. Thus, AMCase is a major transcript in the mouse stomach.

## Supporting Information

Figure S1 **Nucleotide sequence and calculated molecular weight of full-length cDNAs.**
(DOC)

Figure S2 **Real-time PCR quantification of each 10-fold serial a dilution of the full coding cDNA with a known concentration using primer pairs targeting each gene.** The checked DNAs are as follows: A, Chit1; B, AMCase; C, GAPDH; D, β-actin; E, pepsinogen C.
(TIF)

Figure S3 **Relative quantification of Chit1 and AMCase mRNAs levels normalized by GAPDH in mouse tissues.** Relative quantification of Chit1 (A) and AMCase (B) mRNAs in mouse tissues. Both chitinases were quantified by real-time PCR using the standard template DNA. All values obtained were normalized by GAPDH and each relative quantity value is calibrated by the value for testis. C. Ratios of AMCase to Chit1. The upper panel indicates the actual values, whereas the lower panel shows the logarithm of the values.
(TIF)

Figure S4 **Relative quantification of Chit1 and AMCase mRNAs levels normalized by β-actin in mouse tissues.** Relative quantification of Chit1 (A) and AMCase (B) mRNAs in mouse tissues. Both chitinases were quantified by real-time PCR using the standard template DNA. All values obtained were normalized by β-actin each relative quantity value is calibrated by the value for testis. C. Ratios of AMCase to Chit1. The upper panel indicates the actual values, whereas the lower panel shows the logarithm of the values.
(TIF)

Figure S5 **Relative expression of Chit1 and AMCase mRNAs using the ΔΔ Ct method by GAPDH as normalizer in mouse tissues.** Quantification of Chit1 (A) and AMCase (B) mRNAs in mouse tissues. Relative expression levels of both chitinases were quantified by real-time PCR using the ΔΔ Ct method as described in Materials and Methods. We employed GAPDH as a normalizer and each relative quantity value is calibrated by the value for testis. The upper panel indicates the actual values, whereas the lower panel shows the logarithm of the values.
(TIF)

Figure S6 **Relative expression of Chit1 and AMCase mRNAs using the ΔΔ Ct method by β-actin as normalizer in mouse tissues.** Quantification of Chit1 (A) and AMCase (B) mRNAs in mouse tissues. Relative expression levels of both chitinases were quantified by real-time PCR using the ΔΔ Ct method as described in Materials and Methods. We employed β-actin as a normalizer and each relative quantity value is calibrated by the value for testis. The upper panel indicates the actual values, whereas the lower panel shows the logarithm of the values.
(TIF)

Figure S7 **Analysis of Chit1, AMCase and pepsinogen C mRNAs normalized by GAPDH in lung and stomach tissues.** The expression levels of the three genes were determined using the cDNAs prepared from lung (A) or stomach (B) tissues from 3-month-old mice (n = 5) were quantified by real-time PCR using the standard template DNA. All values obtained were normalized by GAPDH. The upper panel indicates the actual values, whereas the lower panel shows the logarithm of each value.
(TIF)

Figure S8 **Analysis of Chit1, AMCase and pepsinogen C mRNAs normalized by β-actin in lung and stomach tissues.** The expression levels of the three genes were determined using the cDNAs prepared from lung (A) or stomach (B) tissues from 3-month-old mice (n = 5) were quantified by real-time PCR using the standard template DNA. All values obtained were normalized by β-actin. The upper panel indicates the actual values, whereas the lower panel shows the logarithm of each value.
(TIF)

Table S1 **The nucleotide sequences of the real-time PCR primers.**
(DOC)

Table S2 **The forward and reverse primers used to construct the standard template DNA.**
(DOC)

Table S3 **Primers for amplifying full coding cDNAs by PCR.**
(DOC)
