# Supplementary material for: Chitinase mRNA Levels by Quantitative PCR Using the Single Standard DNA: Acidic Mammalian Chitinase Is a Major Transcript in the Mouse Stomach
Source: PLoS One. 2012 Nov 21;7(11):e50381. doi: 10.1371/journal.pone.0050381 (PMC3503932; doi:10.1371/journal.pone.0050381)
Supplement: Figure S1 — Nucleotide sequence and calculated molecular weight of full-length cDNAs. (DOC) [file pone.0050381.s001.doc]

**Chit1**

**MW=1002209.8**

CATGGAATTCGGAACAAGTTGTAGAGCTCTCGGCTCCTTCTCCAGCACCAAGTCGGCCTAGACAGTGCCATGCTTCTCACCATGATGATGATGGATTAAACCGCTGAAACTGCCCGTCAGAGGAGAGCAGATCAGGACCTGTGGAGCCAGTCACAGGAGAGCACGGCAGGACCTGTGGAGCGGGTACTCACAGAGCTGATATCCCCAGAGCCTTCATCATGGTGCAGTCCCTGGCCTGGGCAGGTGTGATGACTCTGCTGATGGTCCAGTGGGGCTCTGCTGCAAAACTGGTCTGCTACCTCACCAACTGGTCCCAGTACCGGACGGAGGCAGTTCGGTTCTTTCCCAGGGATGTGGATCCCAACCTGTGTACCCACGTCATCTTTGCTTTTGCTGGAATGGACAACCATCAGCTCAGCACTGTGGAGCACAATGACGAACTTCTCTACCAGGAGCTGAACAGCCTAAAGACTAAGAACCCCAAGCTCAAGACCCTGTTAGCCGTTGGAGGCTGGACCTTTGGTACCCAGAAGTTCACAGACATGGTGGCCACCGCCAGCAACCGGCAGACCTTTGTGAAGTCAGCCCTAAGTTTCCTGCGCACTCAAGGTTTTGATGGCCTTGACCTTGACTGGGAGTTCCCAGGTGGACGTGGGAGCCCCACAGTAGACAAAGAGAGATTCACAGCCCTGATACAGGACTTGGCCAAAGCCTTCCAGGAGGAAGCCCAGTCCTCAGGGAAGGAACGCCTCCTTCTGACTGCAGCTGTACCGAGTGATCGAGGCCTGGTGGATGCTGGCTACGAGGTGGACAAGATTGCCCAGAGCTTGGATTTCATCAACCTTATGGCCTACGACTTCCACAGCTCCTTGGAAAAGACCACAGGGCATAACAGCCCCCTCTACAAAAGGCAAGGAGAAAGTGGGGCAGCCGCTGAGCAAAACGTGGATGCTGCTGTGACGCTCTGGCTGCAGAAGGGGACCCCAGCCAGCAAACTGATCCTTGGCATGCCTACCTATGGACGCTCTTTCACCTTGGCCTCCTCGTCAGACAATGGAGTTGGGGCCCCAGCCACAGGGCCTGGTGCCCCAGGCCCCTATACGAAGGACAAAGGGGTCCTGGCTTACTATGAGGCCTGCTCCTGGAAGGAAAGACACAGAATCGAGGACCAGAAGGTGCCTTACGCCTTCCAGGACAACCAGTGGGTGAGCTTTGACGACGTGGAAAGCTTCAAAGCCAAGGCTGCCTACCTGAAACAGAAGGGGCTGGGAGGAGCCATGGTCTGGGTCCTGGACTTGGATGACTTCAAGGGTTCCTTCTGCAACCAGGGCCCGTACCCTCTCATCCGGACACTACGGCAGGAACTAAATCTTCCATCCGAGACTCCAAGGAGCCCAGAACAGATAATACCTGAGCCACGCCCATCTTCTATGCCAGAGCAGGGACCCAGCCCAGGGCTAGATAACTTCTGCCAAGGCAAAGCTGATGGGGTCTACCCCAACCCTGGAGACGAGTCCACTTACTACAACTGTGGAGGAGGGCGGCTGTTCCAGCAGAGCTGTCCTCCAGGCCTGGTGTTTAGAGCCTCTTGCAAATGTTGTACCTGGAGCGCTCGAGGTCAC

**AMCase**

**MW=903246.8**

CATGGAATTCCATGGAATTCCGGGAGGAACGATGGCCAAGCTACTTCTCGTCACAGGTCTGGCTCTTCTGCTGAATGCTCAGCTGGGGTCTGCCTACAATCTGATATGCTATTTCACCAACTGGGCCCAGTATCGGCCAGGTCTGGGGAGCTTCAAGCCTGATGACATTAACCCCTGCCTGTGTACTCACCTGATCTATGCCTTTGCTGGGATGCAGAACAATGAGATCACCACCATAGAATGGAATGATGTTACTCTCTATAAAGCTTTCAATGACTTGAAAAACAGGAACAGCAAACTGAAAACCCTCCTGGCAATTGGAGGCTGGAACTTTGGAACTGCTCCTTTCACTACCATGGTTTCCACTTCTCAGAACCGCCAGACCTTCATTACCTCAGTCATCAAATTTCTGCGTCAGTATGGGTTTGATGGACTGGACCTGGACTGGGAATACCCAGGCTCACGTGGGAGCCCTCCTCAGGACAAGCATCTCTTCACTGTCCTGGTGAAGGAAATGCGTGAAGCTTTTGAGCAGGAGGCTATTGAGAGCAACAGGCCCAGACTGATGGTTACTGCTGCTGTAGCTGGTGGGATTTCCAACATCCAGGCTGGCTATGAGATCCCTGAACTTTCTAAGTACCTGGATTTCATCCATGTCATGACATATGACCTCCATGGCTCCTGGGAGGGCTACACTGGGGAGAATAGTCCTCTTTACAAATACCCTACTGAGACTGGTAGCAATGCCTACCTCAATGTGGATTATGTCATGAACTATTGGAAGAACAATGGAGCCCCAGCTGAGAAGCTCATTGTTGGATTCCCAGAGTATGGACACACCTTCATCCTGAGAAACCCCTCTGATAATGGAATTGGTGCCCCTACCTCTGGTGATGGCCCTGCTGGGCCCTATACCAGACAGGCTGGGTTCTGGGCCTACTATGAGATTTGCACCTTTCTGAGAAGTGGAGCCACTGAGGTCTGGGATGCCTCCCAAGAAGTGCCCTATGCCTATAAGGCCAACGAGTGGCTTGGCTATGACAATATCAAGAGCTTCAGTGTTAAGGCTCAGTGGCTTAAGCAGAACAATTTTGGAGGTGCCATGATCTGGGCCATTGACCTTGATGACTTCACTGGCTCTTTCTGTGATCAGGGAAAATTTCCTCTGACTTCTACTTTGAACAAAGCCCTTGGCATATCCACTGAAGGTTGCACAGCTCCTGACGTGCCTTCCGAGCCAGTGACTACTCCTCCAGGAAGTGGGAGTGGGGGTGGAAGCTCCGGAGGAAGCTCTGGAGGCAGTGGATTCTGTGCCGACAAAGCAGATGGCCTCTACCCTGTGGCAGATGACAGAAATGCTTTTTGGCAGTGCATCAATGGAATCACATACCAGCAGCATTGTCAAGCAGGGCTTGTTTTTGATACCAGCTGTAATTGCTGCAACTGGCCAGCTCGAGGTCAC

**GAPDH**

**MW=732154.0**

GTGCAGTGCCAGCCTCGTCCCGTAGACAAAATGGTGAAGGTCGGTGTGAACGGATTTGGCCGTATTGGGCGCCTGGTCACCAGGGCTGCCATTTGCAGTGGCAAAGTGGAGATTGTTGCCATCAACGACCCCTTCATTGACCTCAACTACATGGTCTACATGTTCCAGTATGACTCCACTCACGGCAAATTCAACGGCACAGTCAAGGCCGAGAATGGGAAGCTTGTCATCAACGGGAAGCCCATCACCATCTTCCAGGAGCGAGACCCCACTAACATCAAATGGGGTGAGGCCGGTGCTGAGTATGTCGTGGAGTCTACTGGTGTCTTCACCACCATGGAGAAGGCCGGGGCCCACTTGAAGGGTGGAGCCAAAAGGGTCATCATCTCCGCCCCTTCTGCCGATGCCCCCATGTTTGTGATGGGTGTGAACCACGAGAAATATGACAACTCACTCAAGATTGTCAGCAATGCATCCTGCACCACCAACTGCTTAGCCCCCCTGGCCAAGGTCATCCATGACAACTTTGGCATTGTGGAAGGGCTCATGACCACAGTCCATGCCATCACTGCCACCCAGAAGACTGTGGATGGCCCCTCTGGAAAGCTGTGGCGTGATGGCCGTGGGGCTGCCCAGAACATCATCCCTGCATCCACTGGTGCTGCCAAGGCTGTGGGCAAGGTCATCCCAGAGCTGAACGGGAAGCTCACTGGCATGGCCTTCCGTGTTCCTACCCCCAATGTGTCCGTCGTGGATCTGACGTGCCGCCTGGAGAAACCTGCCAAGTATGATGACATCAAGAAGGTGGTGAAGCAGGCATCTGAGGGCCCACTGAAGGGCATCTTGGGCTACACTGAGGACCAGGTTGTCTCCTGCGACTTCAACAGCAACTCCCACTCTTCCACCTTCGATGCCGGGGCTGGCATTGCTCTCAATGACAACTTTGTCAAGCTCATTTCCTGGTATGACAATGAATACGGCTACAGCAACAGGGTGGTGGACCTCATGGCCTACATGGCCTCCAAGGAGTAAGAAACCCTGGACCACCCACCCCAGCAAGGACACTGAGCAAGAGAGGCCCTATCCCAACTCGGCCCCCAACACTGAGCATCTCCCTCACAATTTCCATCCCAGACCCCCATAATAACAGGAGGGGCCTAGGGAGCCCTCCCTACTCTCTTGAAT

**β-actin**

**MW=979274.0**

GCGTCCACCCGCGAGCACAGCTTCTTTGCAGCTCCTTCGTTGCCGGTCCACACCCGCCACCAGTTCGCCATGGATGACGATATCGCTGCGCTGGTCGTCGACAACGGCTCCGGCATGTGCAAAGCCGGCTTCGCGGGCGACGATGCTCCCCGGGCTGTATTCCCCTCCATCGTGGGCCGCCCTAGGCACCAGGGTGTGATGGTGGGAATGGGTCAGAAGGACTCCTATGTGGGTGACGAGGCCCAGAGCAAGAGAGGTATCCTGACCCTGAAGTACCCCATTGAACATGGCATTGTTACCAACTGGGACGACATGGAGAAGATCTGGCACCACACCTTCTACAATGAGCTGCGTGTGGCCCCTGAGGAGCACCCTGTGCTGCTCACCGAGGCCCCCCTGAACCCTAAGGCCAACCGTGAAAAGATGACCCAGATCATGTTTGAGACCTTCAACACCCCAGCCATGTACGTAGCCATCCAGGCTGTGCTGTCCCTGTATGCCTCTGGTCGTACCACAGGCATTGTGATGGACTCCGGAGACGGGGTCACCCACACTGTGCCCATCTACGAGGGCTATGCTCTCCCTCACGCCATCCTGCGTCTGGACCTGGCTGGCCGGGACCTGACAGACTACCTCATGAAGATCCTGACCGAGCGTGGCTACAGCTTCACCACCACAGCTGAGAGGGAAATCGTGCGTGACATCAAAGAGAAGCTGTGCTATGTTGCTCTAGACTTCGAGCAGGAGATGGCCACTGCCGCATCCTCTTCCTCCCTGGAGAAGAGCTATGAGCTGCCTGACGGCCAGGTCATCACTATTGGCAACGAGCGGTTCCGATGCCCTGAGGCTCTTTTCCAGCCTTCCTTCTTGGGTATGGAATCCTGTGGCATCCATGAAACTACATTCAATTCCATCATGAAGTGTGACGTTGACATCCGTAAAGACCTCTATGCCAACACAGTGCTGTCTGGTGGTACCACCATGTACCCAGGCATTGCTGACAGGATGCAGAAGGAGATTACTGCTCTGGCTCCTAGCACCATGAAGATCAAGATCATTGCTCCTCCTGAGCGCAAGTACTCTGTGTGGATCGGTGGCTCCATCCTGGCCTCACTGTCCACCTTCCAGCAGATGTGGATCAGCAAGCAGGAGTACGATGAGTCCGGCCCCTCCATCGTGCACCGCAAGTGCTTCTAGGCGGACTGTTACTGAGCTGCGTTTTACACCCTTTCTTTGACAAAACCTAACTTGCGCAGAAAAAAAAAAAATAAGAGACAACATTGGCATGGCTTTGTTTTTTTAAATTTTTTTTAAAGTTTTTTTTTTTTTTTTTTTTTTTTTTTTTAAGTTTTTTTGTTTTGTTTTGGCGCTTTTGACTCAGGATTTAAAAACTGGAACGGTGAAGGCGACAGCAGTTGGTTGGAGCAAACATCCCCCAAAGTTCTACAAATGTGGCTGAGGACTTTGTACATTGTTTTGTTTTTTTTTTTTTTTGGTTTTGTCTTTTTTTAATAGTCATTCCAAGTATCCATGAAATAAGTGGTTACAGGAAGTCCCTCACCCTCCCAAAAGCCACCCCCACTCC

**Pepsinogen C**

**MW=803236.0**

GCTCGAAGTGATTTCTTCCAGTGAGACCAGCTGCAAACCGGCATCATGAAGTGGATGGTGGTCGCCTTGCTCTGCCTCCCACTCCTGGAGGCAGCTTTGATCAGGGTCCCCCTGAAGAAAATGAAGAGTATCCGGGAGACCATGAAGGAACAAGGTGTCCTCAAAGACTTTCTGAAGAACCACAAGTATGACCCTGGCCAGAAATACCACTTTGGCAAGTTTGGTGACTACAGTGTACTCTATGAGCCCATGGCCTATATGGATGCTTCCTACTATGGTGAGATCAGCATCGGGACTCCACCCCAGAACTTCCTGGTCCTTTTCGACACTGGCTCCTCCAACCTGTGGGTGTCTTCTGTCTACTGCCAGAGCGAGGCCTGCACCACACACACCCGCTACAACCCCAGCAAGTCCTCCACCTACTACACTCAAGGGCAGACCTTCTCCCTGCAGTACGGCACCGGCAGCCTTACCGGCTTCTTCGGCTATGACACTCTGAGAGTCCAAAGCATCCAGGTCCCTAACCAGGAGTTCGGCCTGAGTGAGAATGAGCCTGGCACCAATTTTGTCTACGCCCAATTTGACGGGATCATGGGCCTGGCCTACCCCGGCCTGTCTTCAGGGGGCGCCACCACCGCCTTGCAGGGCATGTTGGGGGAGGGCGCTCTGTCCCAGCCCCTCTTCGGTGTCTACCTTGGCAGCCAGCAGGGGTCTAACGGCGGGCAGATTGTGTTCGGTGGCGTGGACGAGAACCTGTACACTGGCGAGCTCACCTGGATTCCTGTCACCCAGGAGCTTTACTGGCAGATCACCATTGACGACTTCCTTATTGGCAACCAGGCCTCTGGCTGGTGCTCCTCCTCTGGCTGCCAAGGCATTGTAGACACAGGCACCTCTCTGCTCGTCATGCCTGCCCAGTACCTGAATGAACTTCTGCAGACCATAGGAGCCCAGGAAGGAGAGTATGGACAGTATTTTGTGAGCTGCGACAGCGTCAGTAGCCTGCCTACCCTCACTTTTGTCCTCAATGGTGTCCAGTTCCCCCTGTCACCCTCTTCCTACATCATCCAGGAGGAAGGCTCCTGCATGGTGGGTCTGGAGAGCCTCTCCCTGAACGCTGAGAGTGGCCAGCCCCTCTGGATCCTCGGGGATGTCTTCCTCAGGTCTTACTATGCTGTCTTCGACATGGGCAATAACAGGGTGGGCCTTGCCCCTTCTGTCTAGACTTGACATCTAGACAAACCCCCCCCCCACCCCCGTGCCCTTCCTTCTTCCCCTCAGCCTGTCCCATCTGCCCCGC
