# Supplementary figures and images for: Chitinase mRNA Levels by Quantitative PCR Using the Single Standard DNA: Acidic Mammalian Chitinase Is a Major Transcript in the Mouse Stomach
Source: PLoS One. 2012 Nov 21;7(11):e50381. doi: 10.1371/journal.pone.0050381 (PMC3503932; doi:10.1371/journal.pone.0050381)

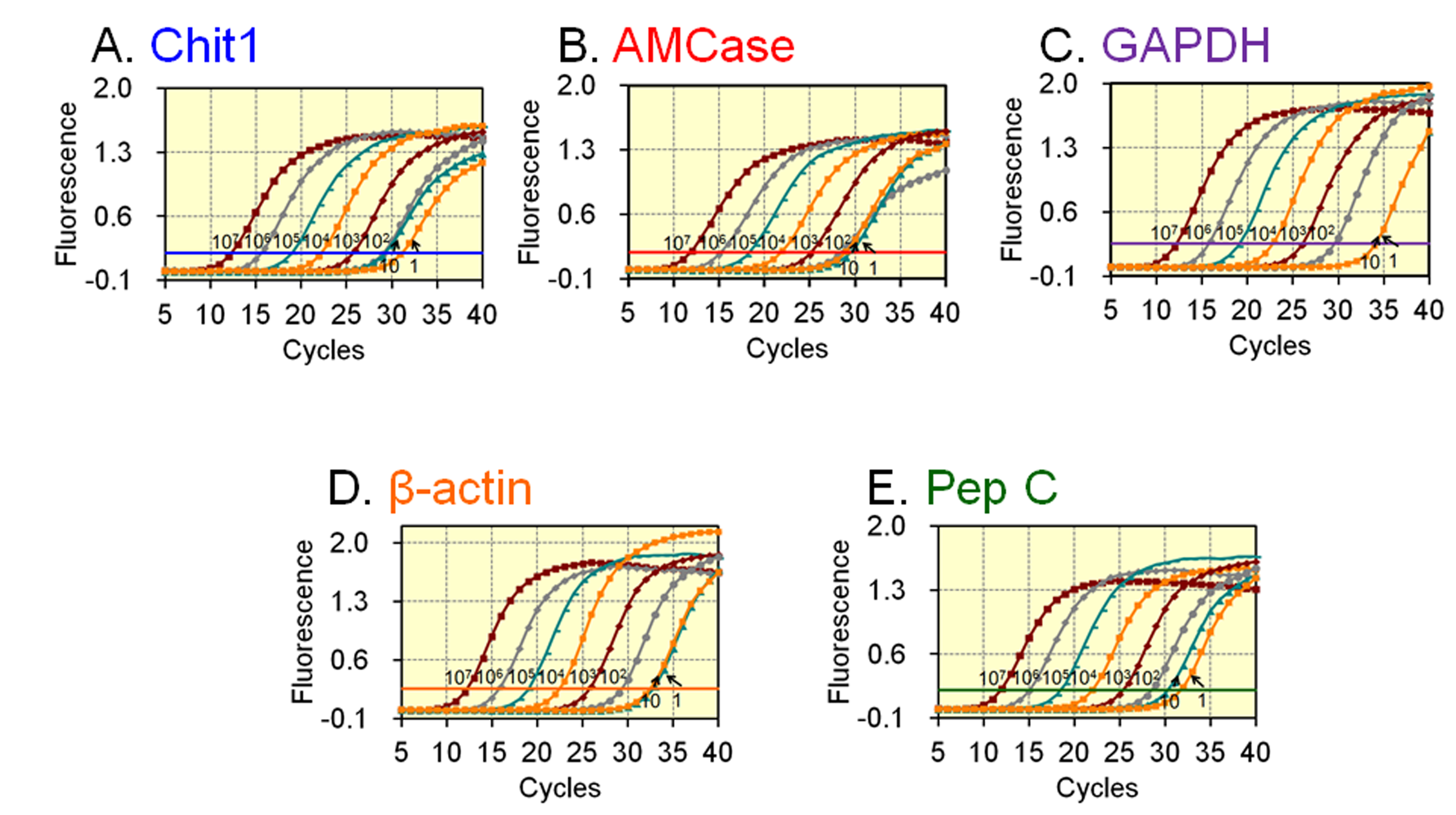

Supplement: Figure S2 — Real-time PCR quantification of each 10-fold serial a dilution of the full coding cDNA with a known concentration using primer pairs targeting each gene. The checked DNAs are as follows: A, Chit1; B, AMCase; C, GAPDH; D, β-actin; E, pepsinogen C. (TIF) [file pone.0050381.s002.tif]

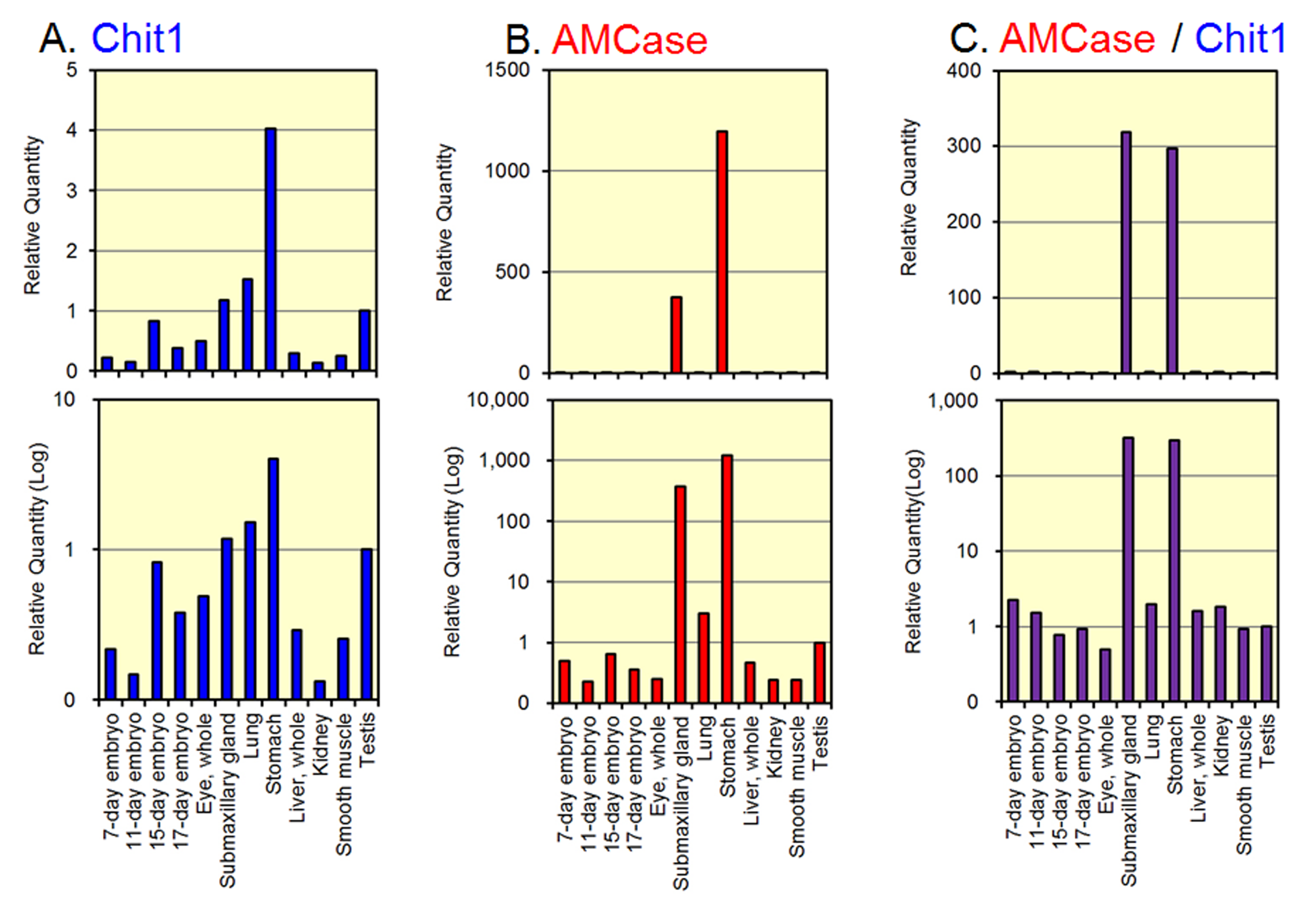

Supplement: Figure S3 — Relative quantification of Chit1 and AMCase mRNAs levels normalized by GAPDH in mouse tissues. Relative quantification of Chit1 (A) and AMCase (B) mRNAs in mouse tissues. Both chitinases were quantified by real-time PCR using the standard template DNA. All values obtained were normalized by GAPDH and each relative quantity value is calibrated by the value for testis. C. Ratios of AMCase to Chit1. The upper panel indicates the actual values, whereas the lower panel shows the logarithm of the values. (TIF) [file pone.0050381.s003.tif]

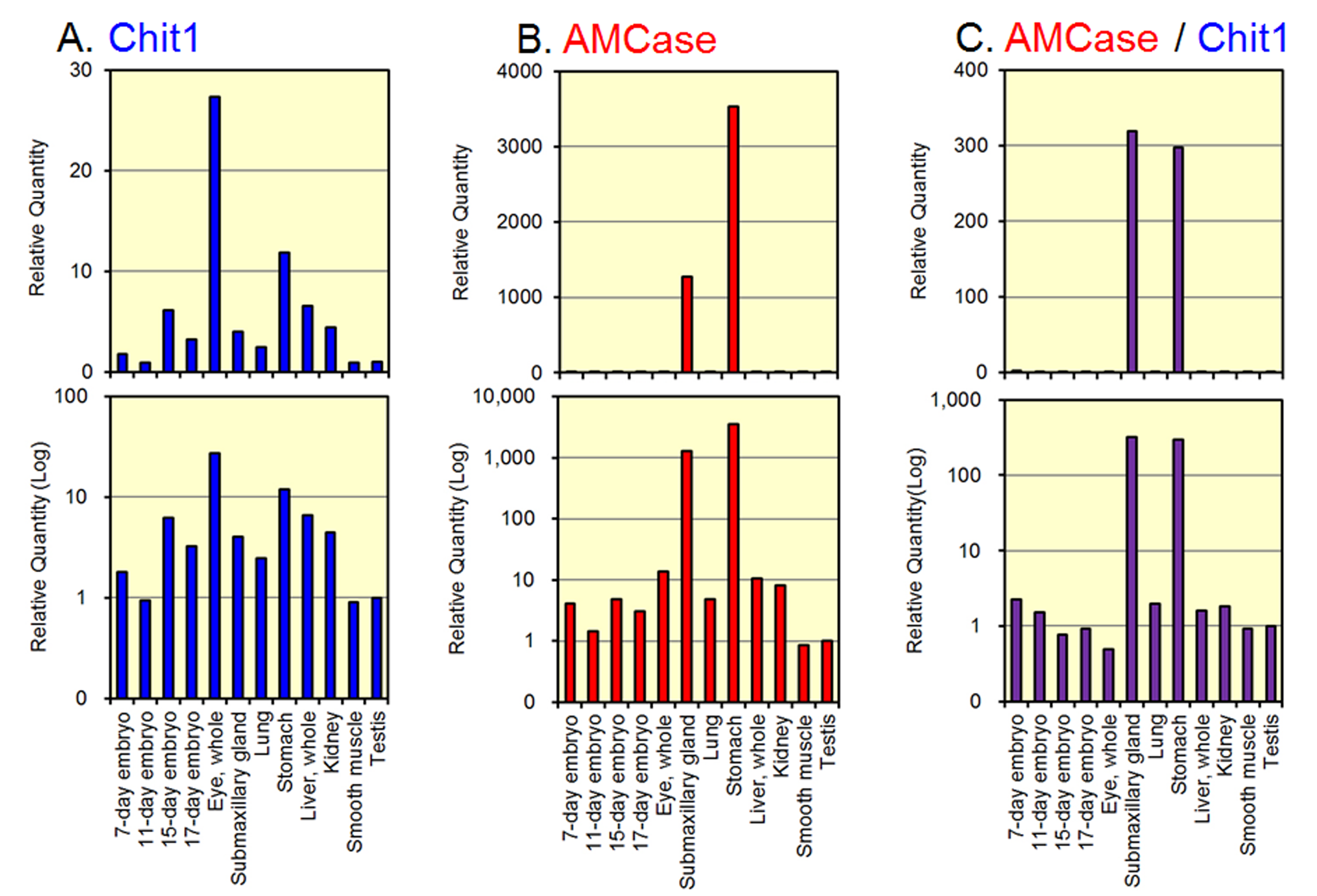

Supplement: Figure S4 — Relative quantification of Chit1 and AMCase mRNAs levels normalized by β-actin in mouse tissues. Relative quantification of Chit1 (A) and AMCase (B) mRNAs in mouse tissues. Both chitinases were quantified by real-time PCR using the standard template DNA. All values obtained were normalized by β-actin each relative quantity value is calibrated by the value for testis. C. Ratios of AMCase to Chit1. The upper panel indicates the actual values, whereas the lower panel shows the logarithm of the values. (TIF) [file pone.0050381.s004.tif]

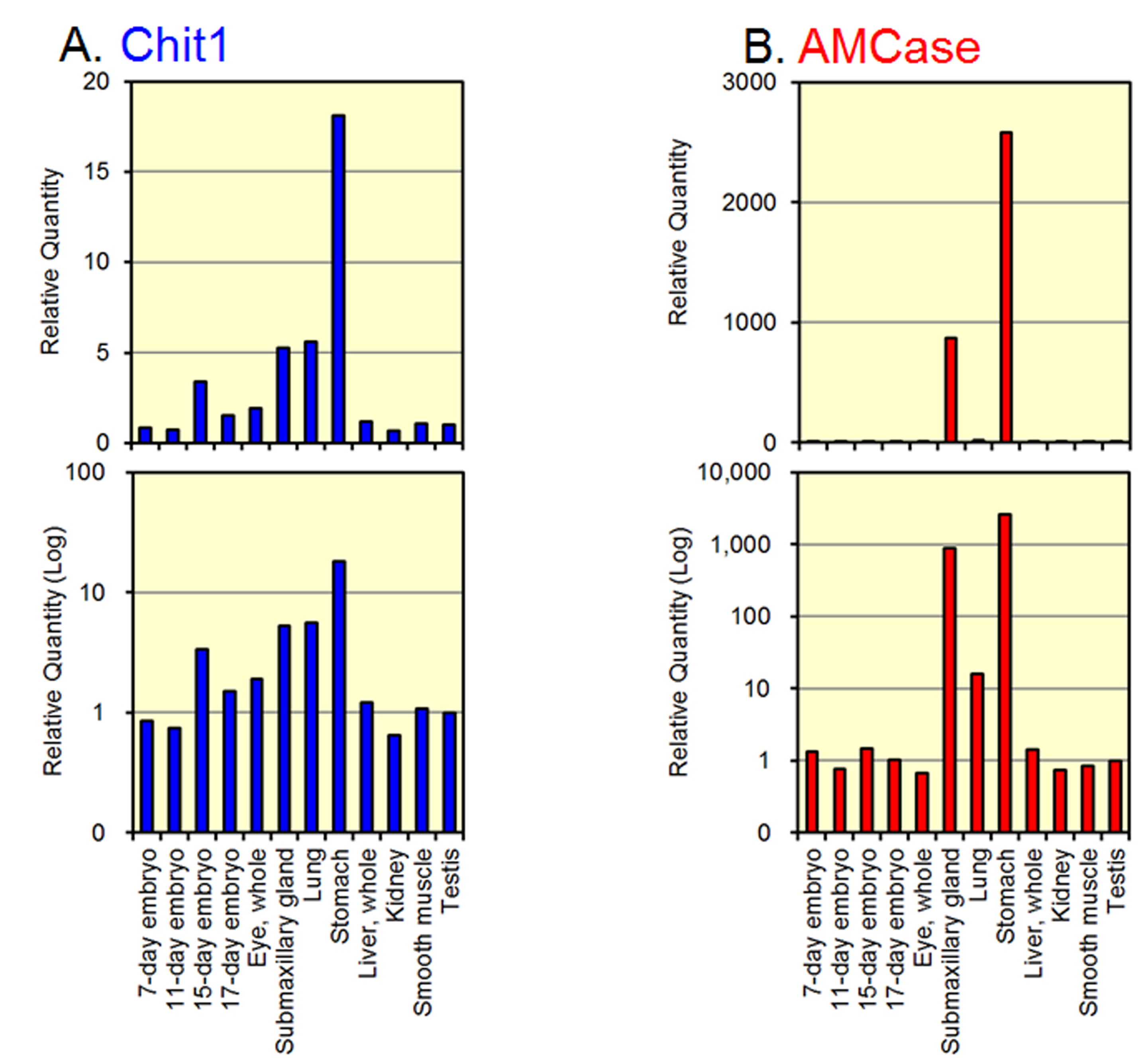

Supplement: Figure S5 — Relative expression of Chit1 and AMCase mRNAs using the ΔΔ Ct method by GAPDH as normalizer in mouse tissues. Quantification of Chit1 (A) and AMCase (B) mRNAs in mouse tissues. Relative expression levels of both chitinases were quantified by real-time PCR using the ΔΔ Ct method as described in Materials and Methods. We employed GAPDH as a normalizer and each relative quantity value is calibrated by the value for testis. The upper panel indicates the actual values, whereas the lower panel shows the logarithm of the values. (TIF) [file pone.0050381.s005.tif]

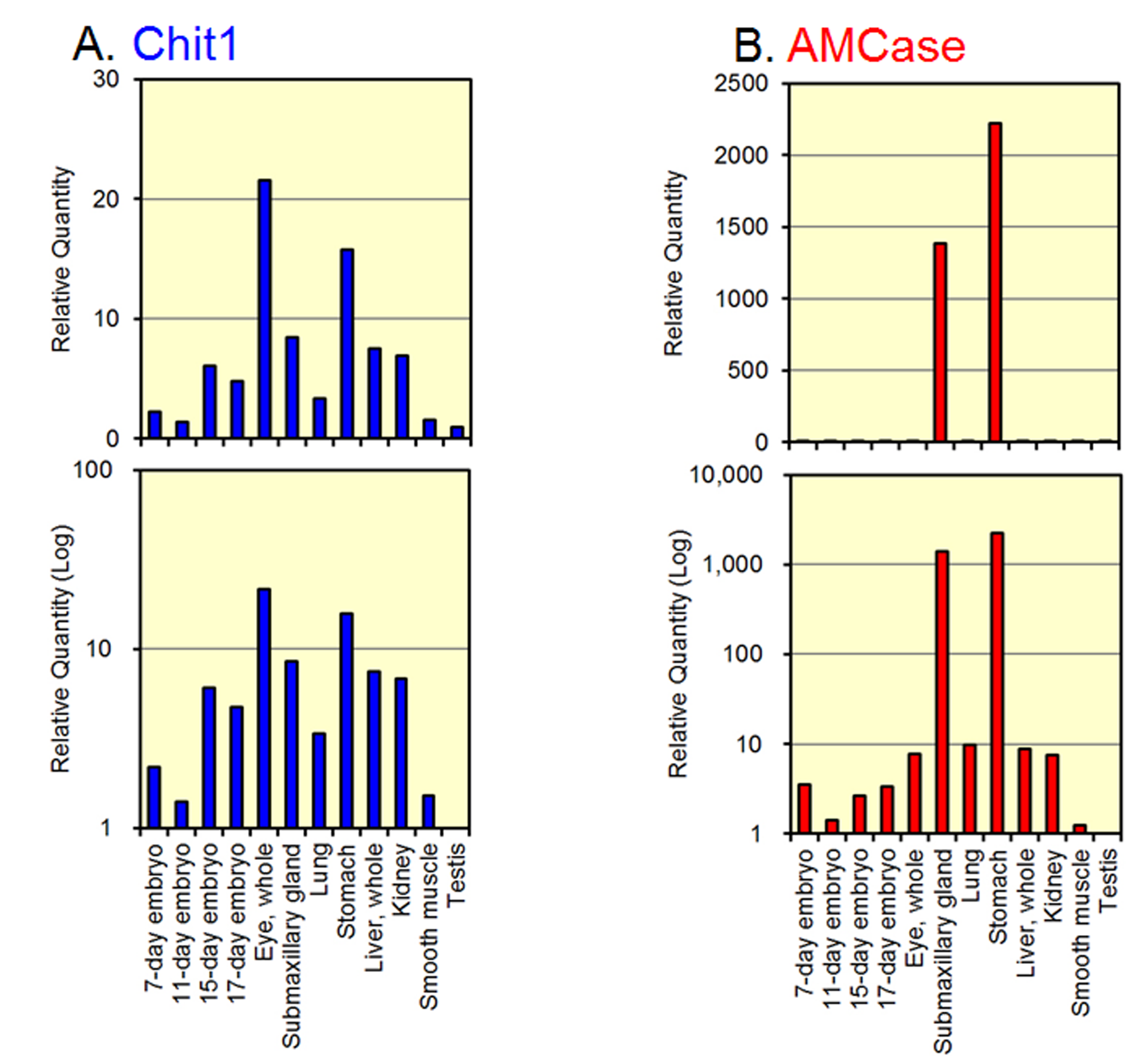

Supplement: Figure S6 — Relative expression of Chit1 and AMCase mRNAs using the ΔΔ Ct method by β-actin as normalizer in mouse tissues. Quantification of Chit1 (A) and AMCase (B) mRNAs in mouse tissues. Relative expression levels of both chitinases were quantified by real-time PCR using the ΔΔ Ct method as described in Materials and Methods. We employed β-actin as a normalizer and each relative quantity value is calibrated by the value for testis. The upper panel indicates the actual values, whereas the lower panel shows the logarithm of the values. (TIF) [file pone.0050381.s006.tif]

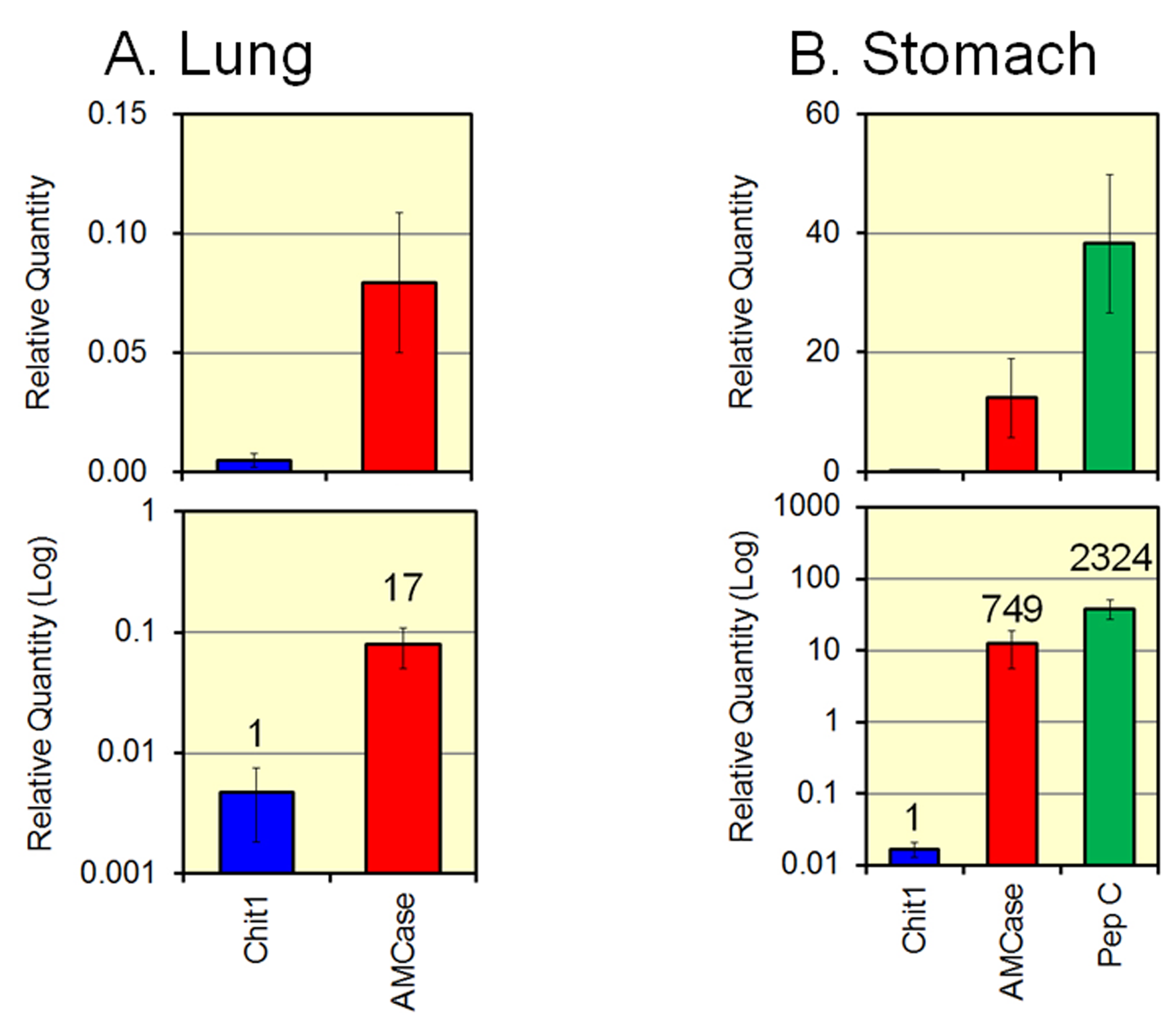

Supplement: Figure S7 — Analysis of Chit1, AMCase and pepsinogen C mRNAs normalized by GAPDH in lung and stomach tissues. The expression levels of the three genes were determined using the cDNAs prepared from lung (A) or stomach (B) tissues from 3-month-old mice (n = 5) were quantified by real-time PCR using the standard template DNA. All values obtained were normalized by GAPDH. The upper panel indicates the actual values, whereas the lower panel shows the logarithm of each value. (TIF) [file pone.0050381.s007.tif]

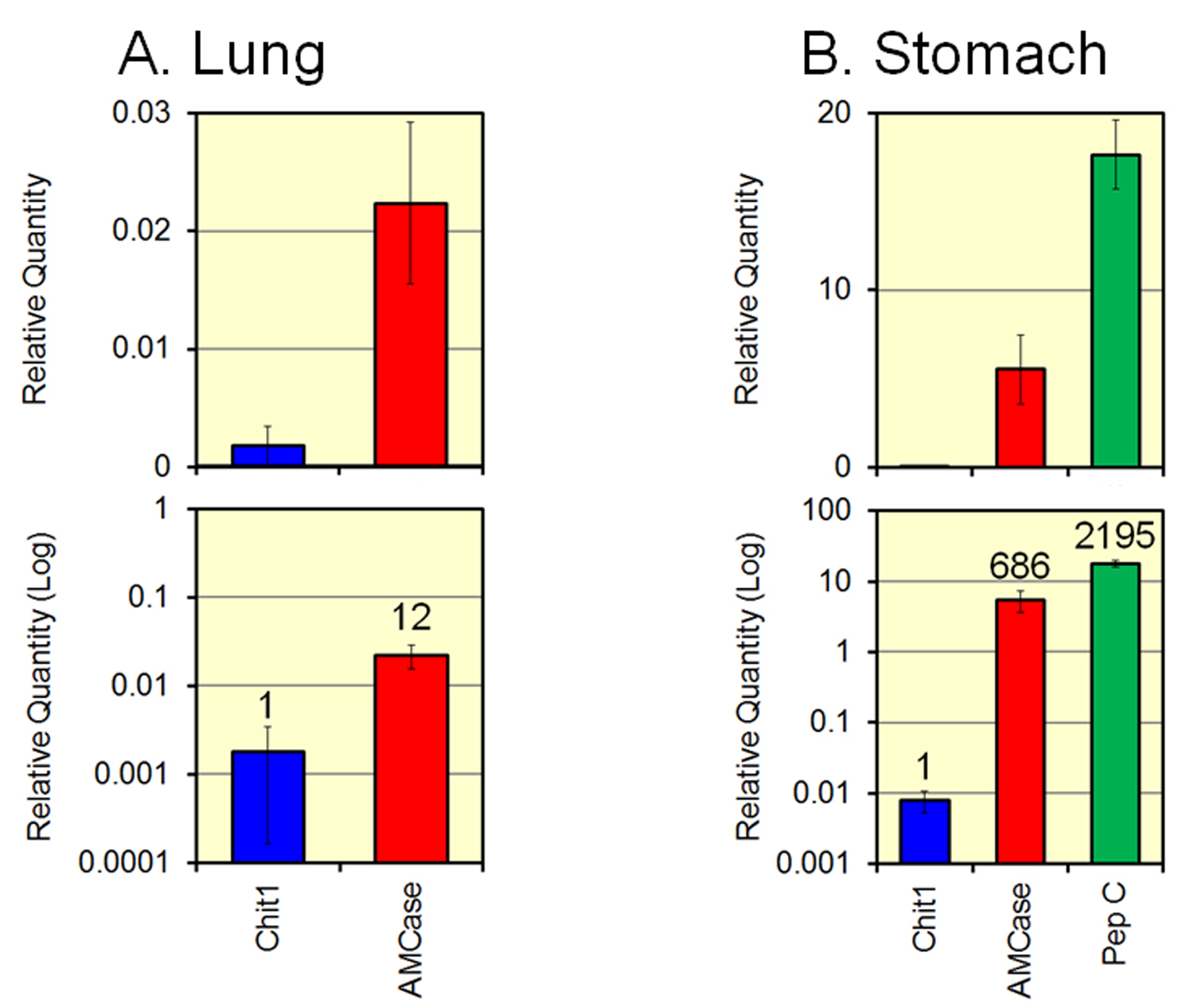

Supplement: Figure S8 — Analysis of Chit1, AMCase and pepsinogen C mRNAs normalized by β-actin in lung and stomach tissues. The expression levels of the three genes were determined using the cDNAs prepared from lung (A) or stomach (B) tissues from 3-month-old mice (n = 5) were quantified by real-time PCR using the standard template DNA. All values obtained were normalized by β-actin. The upper panel indicates the actual values, whereas the lower panel shows the logarithm of each value. (TIF) [file pone.0050381.s008.tif]
