# Supplementary material for: Chitinase mRNA Levels by Quantitative PCR Using the Single Standard DNA: Acidic Mammalian Chitinase Is a Major Transcript in the Mouse Stomach
Source: PLoS One. 2012 Nov 21;7(11):e50381. doi: 10.1371/journal.pone.0050381 (PMC3503932; doi:10.1371/journal.pone.0050381)
Supplement: Table S1 — The nucleotide sequences of the real-time PCR primers. (DOC) [file pone.0050381.s009.doc]

AMCase_Fw: TTTTGGCAGTGCATCAATGG

AMCase_Rv: GCAGCAATTACAGCTGGTATCAA

Chit1_Fw: CGGCAGGAACTAAATCTTCCAT

Chit1_Rv: TGGGCGTGGCTCAGGTAT

Pep C_Fw: TGCCAAGGCATTGTAGACACA

Pep C_Rv: CTCCTATGGTCTGCAGAAGTTCATT

GAPDH_Fw: TGTGTCCGTCGTGGATCTGA

GAPDH_Rv: CCTGCTTCACCACCTTCTTGA

β-Actin_Fw: ACGGCCAGGTCATCACTATTG

β-Actin_Rv: CAAGAAGGAAGGCTGGAAAAGA
