# Supplementary material for: Chitinase mRNA Levels by Quantitative PCR Using the Single Standard DNA: Acidic Mammalian Chitinase Is a Major Transcript in the Mouse Stomach
Source: PLoS One. 2012 Nov 21;7(11):e50381. doi: 10.1371/journal.pone.0050381 (PMC3503932; doi:10.1371/journal.pone.0050381)
Supplement: Table S2 — The forward and reverse primers used to construct the standard template DNA. (DOC) [file pone.0050381.s010.doc]

Quant_AMC_Fw: CATGGAATTCGGAGGCAGTGGATTCTGTGCCGACA

Bgl_quant_AMC_Rv: TGACAGATCTTGGCCAGTTGCAGCAATTACAGCTG

Bgl_quant_Pep C_Fw: CATGAGATCTTGGCAACCAGGCCTCTGGCTGGTGC

Xho_quant_Pep C_Rv: TGACCTCGAGACAAAATACTGTCCATACTCTCCTT

Xho_quant_Chit1_Fw: CATGCTCGAGTGGACTTGGATGACTTCAAGGGTTC

Pst_quant_Chit1_Rv: TGACCTGCAGTAGCCCTGGGCTGGGTCCCTGCTCT

Pst_quant_GAPDH_Fw: TGACCTGCAGGAGCTGAACGGGAAGCTCACTGGCA

Not_quant_GAPDH_Rv: TCGAGCGGCCGCTCCTCAGTGTAGCCCAAGATGCC

Not_quant_β-Actin_Fw: TCGAGCGGCCGCCGAGCAGGAGATGGCCACTGCCG

Quant_β-Actin_Rv: TGACAGATCTTGGGTACATGGTGGTACCACCAGAC
