# Supplementary material for: Chitinase mRNA Levels by Quantitative PCR Using the Single Standard DNA: Acidic Mammalian Chitinase Is a Major Transcript in the Mouse Stomach
Source: PLoS One. 2012 Nov 21;7(11):e50381. doi: 10.1371/journal.pone.0050381 (PMC3503932; doi:10.1371/journal.pone.0050381)
Supplement: Table S3 — Primers for amplifying full coding cDNAs by PCR. (DOC) [file pone.0050381.s011.doc]

Full_AMCase_FW: CATGGAATTCCGGGAGGAACGATGGCCAAGCTACT

Full_AMCase_RV: GTGACCTCGAGCTGGCCAGTTGCAGCAATTACAGC

Full_Chit1_Fw: CATGGAATTCGGAACAAGTTGTAGAGCTCTCGGCT

Full_Chit1_RV: GTGACCTCGAGCGCTCCAGGTACAACATTTGCAAG

Full_Pep C_Fw: GCTCGAAGTGATTTCTTCCAGTGAG

Full_Pep C Rv: GCGGGGCAGATGGGACAGGCTGAGG

Full_GAPDH_Fw: GTGCAGTGCCAGCCTCGTCCCGTAG

Full_GAPDH_Rv: ATTCAAGAGAGTAGGGAGGGCTCCC

Full_β-Actin_Fw: GCGTCCACCCGCGAGCACAGCTTCT

Full_β-Actin_Rv: GGAGTGGGGGTGGCTTTTGGGAGGG
